# Pneumatosis intestinalis a trap for the unwary: Case series and literature review

**DOI:** 10.1016/j.ijscr.2018.10.079

**Published:** 2018-11-02

**Authors:** Sunny Dhadlie, Daniel Mehanna, James McCourtney

**Affiliations:** a Caboolture Hospital, 120 McKean Street, 4510, Queenland, Australia; b Royal Alexandra Hospital, Corsebar Rd, Paisely, PA2 9PN, Scotland, UK

**Keywords:** Pneumatosis intestinalis, Pneumatosis cystoids intestinalis

## Abstract

**Introduction:**

Pneumatosis Intestinalis (PI) can present with a broad range of presentations from chronic and non-specific to acute and life threatening. It is paradoxically one of the few conditions where a pneumoperitoneum found in the diagnostic workup is not necessarily an indication for laparotomy.

**Presentation of cases:**

The first case is of a 75 year old gentlemen who attended the emergency department after several weeks of worsening abdominal pain and weight loss. At laparotomy, two segments of nodular, abnormal-looking small bowel were identified with bubbles of air seen in the small bowel mesentery.

The second case is of an 86 year old man with of a 5-month history of abdominal pain, weight loss, nausea and diarrhoea. A subsequent endoscopy revealed diffuse gastritis with a small antral gastric ulcer with a small amount of blood. The CT scan demonstrated free air within the bowel wall, with a follow up scan performed 3 weeks later revealing an increase in the amount of free air.

**Discussion:**

PI is a rare condition characterised by the presence of subserosal and submucosal gas filled cysts occurring anywhere in the gastrointestinal tract from the oesophagus to the anus.

Numerous conditions have been associated with PI including bowel obstruction, infections, ischemia and there have been reported cases suggesting as association with Crohn’s disease.

**Conclusion:**

PI is a condition that may manifest with a wide range of symptoms from mild abdominal pain to acute peritonitis. Its treatment is generally medical and even with radiological evidence of perforation laparotomy may not be indicated if the patient is clinically well.

## Highlights

• Pneumatosis Intestinalis is a rare condition that may manifest with a wide range of symptoms from mild abdominal pain to acute peritonitis.
• In general, surgery is only indicated in symptomatic patients where medical therapy has failed and in patients presenting acutely unwell with a surgical abdomen.
• Treatment is generally medical and even with radiological evidence of perforation laparotomy may not be indicated if the patient is clinically well.
• Treatment with oxygen or hyperbaric oxygen has been shown to be effective.

## 1 Introduction

PI is a rare condition characterised by the presence of gas filled cysts in the gastrointestinal tract. Its treatment depends on the acuity of the presentation and any underlying conditions and may present with a “benign pneumoperitoneum” not necessitating surgery.

## 2 Presentation of cases

These cases have been reported in line with the PROCESS criteria [1] researchregistry4407

### 2.1 Case 1

A 75 year old retired electrician presented to the emergency department with a several week history of worsening abdominal pain and weight loss. He had a background of ischemic heart disease, type 2 diabetes, chronic obstructive airways disease and a duodenal ulcer diagnosed by endoscopy 2 years previously.

On presentation he was dehydrated but haemodynamically stable and afebrile. His abdomen was soft, with general tenderness in the epigastrium with mild rebound but no rigidity. Full blood count, electrolytes and coagulation studies were all normal. A chest x-ray showed free air underneath the left hemi diaphragm.

**Fig. 1:**
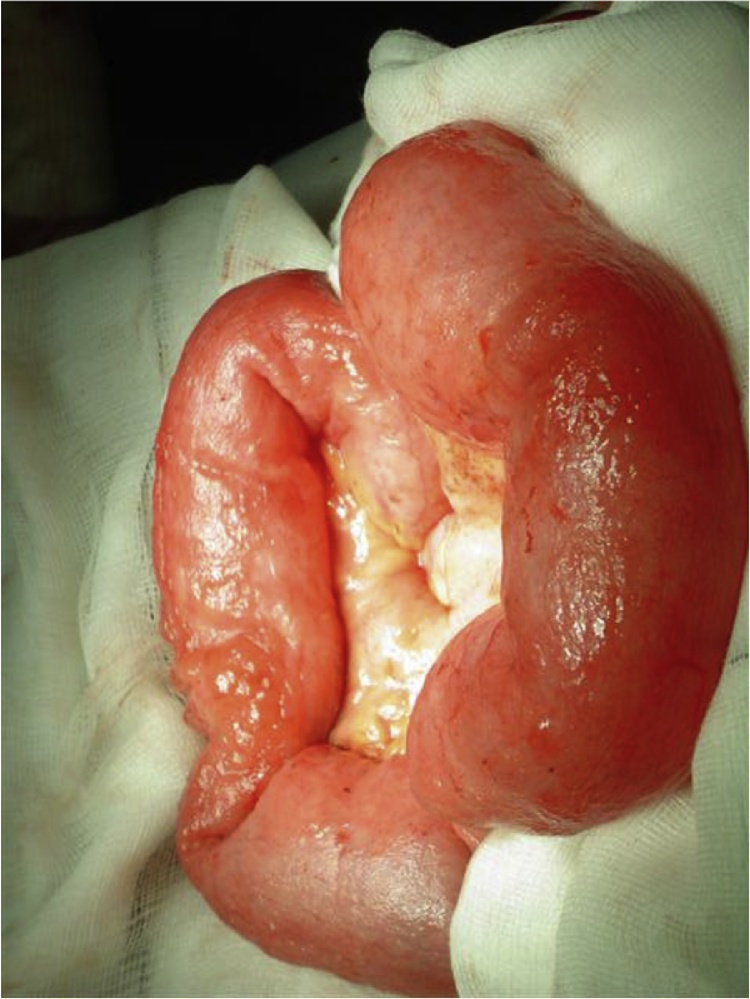
Small bowel segment with bubble of air.

The diagnosis of a perforated viscous was made, thought to be due to a perforated peptic ulcer and he was resuscitated and taken to theatre for laparotomy. At laparotomy two segments of nodular, abnormal-looking small bowel were identified, the first in the proximal jejunum and the second in the distal jejunum (Fig. 1). In the proximal segment bubbles of air were seen in the small bowel mesentery suggesting a small bowel perforation along the mesenteric border of the bowel. A small bowel resection was performed with a hand sewn end-to-end anastomosis. The lesser sac was entered, the duodenum mobilized and small bowel examined to exclude another site of perforation.

He was transferred to the high dependency unit for post-operative management where he developed acute renal failure, which responded to attention to fluid management. On the 12th post-operative day he developed increasing abdominal distension with subsequent plain films and a CT scan performed to exclude an anastomotic leak and collection. A gastograffin small bowel study showed a dilated oesophagus as well as generally dilated small bowel. He was treated conservatively and made a slow recovery. He was discharged on the 27th post operative day.

He subsequently represented a month later with recurrent vomiting and was admitted for investigation. A CT scan showed the presence of locules of free air, as he did not demonstrate signs of peritonism he was treated conservatively with parental nutrition, oral oxygen therapy and intravenous antibiotics to treat the underlying pneumatosis and was then discharged 26 days later.

**Fig. 2:**
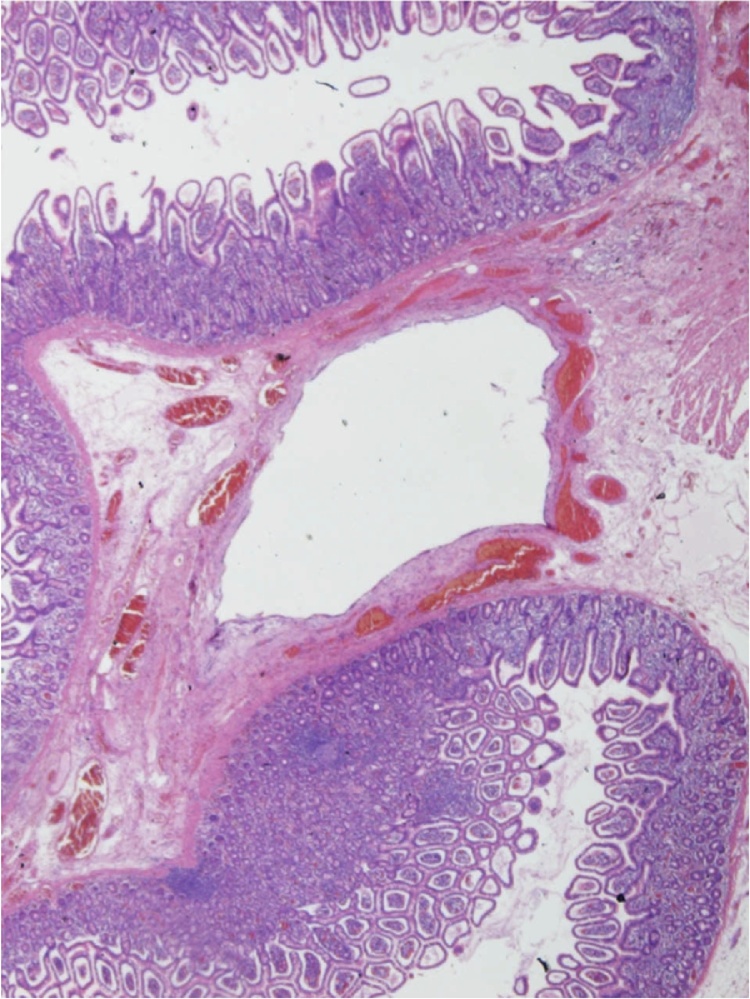
Low power view of submucosal cysts.

**Fig. 3:**
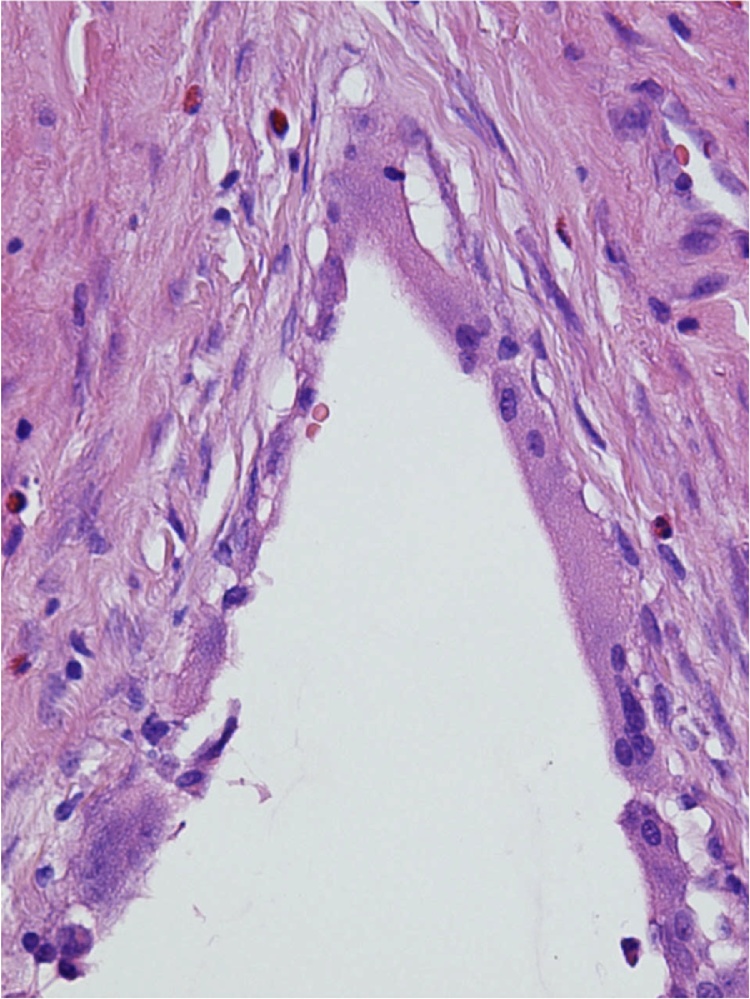
High power view of cyst lining demonstrating giant cells and histiocytes.

During the first admission to hospital, histological examination was performed on sections from the jejunal resection specimen taken at laparotomy. The striking diagnostic feature of multiple submucosal cysts appearing as large ‘empty’ spaces (Fig. 2) lined by an histiocytic inflammatory infiltrate which includes giant cells (Fig. 3). The appearances are characteristic of Pneumatosis Cystoides Intestinalis. Low-grade acute peritonitis was also present, consistent with the clinical impression of perforation, although no site was identified it was felt perforation most likely resulted from rupture of one of these cysts.

### 2.2 Case 2

An 86 year old man was referred for consultation in the surgical outpatients clinic regarding investigation of a 5 month history of abdominal pain, weight loss, nausea and diarrhoea.

He had a CT scan prior to referral demonstrating a small amount of ascitic fluid.

The patient had no past medical history of note. His last endoscopy and colonoscopy were 10 years ago, with the colonoscopy identifying a tubular adenoma and hyperplastic polyps in rectosigmoid region.

On review he had observations within normal parameters and his abdominal examination was normal.

A subsequent endoscopy revealed diffuse gastritis with a small antral gastric ulcer with a small amount of blood. Helicobacter pylori testing was negative. On colonoscopy there was moderate sigmoid diverticular disease. Three polyps were removed from the ascending colon, sigmoid colon and rectum. The patient was commenced of a proton pump inhibitor and review in rooms arranged for 4 weeks.

On review at 4 weeks the patient reported a complete resolution of symptoms that he had prior to initial consultation. His only complaint was of slightly more flatus than usual.

Abdominal examination was again unremarkable. A follow up endoscopy was arranged which was normal.

A routine abdominal CT scan was arranged to assess the presence of residual free fluid that was seen on the CT scan performed prior to the patient’s referral.

The CT scan demonstrated free air within the bowel wall, with a follow up scan performed 3 weeks later revealing an increase in the amount of free air. The patient had a barium swallow, which was negative for a leak.

The patient was referred to a tertiary centre for management, hyperbaric therapy was considered but ultimately the patient was treated conservatively with oral oxygen therapy and antibiotics.

## 3 Discussion

PI is a rare condition characterised by the presence of subserosal and submucosal gas filled cysts occurring anywhere in the gastrointestinal tract from the oesophagus to the anus. [2] Although it was first described pathologically in 1730 by DuVernoi it was not until 1835 that Mayer coined the term “pneumatosis cystoides intestinorum” to describe this condition [3]. Although PI is described under many names, including pneumatosis cystoides intestinalis, intestinal emphysema and intestinal gas cysts, most authors term the benign form “pneumatosis cystoides intestinalis”. [4]

In a large study of 919 cases, Jamart found that it had a peak incidence between the ages of 41 and 50 years and was more common in males with a male to female ratio of 3:1. The small intestine was found to be the most commonly affected site (42%) followed by the large intestine (36%). In 22% both the small and large intestine were affected. [5]

The pathological findings are that of multiple gas filled cysts in a sessile or a pedunculated form usually found in a submucosal or subserosal position. Numerous conditions have been associated with PI including bowel obstruction, infections, ischemia and there have been reported cases suggesting as association with Crohn’s disease. [[6], [7], [8]] In additional numerous non-surgical conditions including collagen vascular diseases and respiratory diseases (COPD, asthma and cystic fibrosis) have been found to be associated with PI [9]. The majority of patients with collagen vascular disease and PI have gastrointestinal hypomotility and often have intestinal pseudo-obstruction [10].

Pneumatosis intestinalis can be classified into a primary or idiopathic form (15% of cases) and a secondary form. The pathogenesis of PI is unknown however several theories exit. The three most common are the mechanical, pulmonary and bacterial theories. [[11], [12], [13], [14]] The mechanical theory suggests that in the presence of significant intraluminal pressure gas penetrates through the mucosa breaking into the submucosa or subserosa via lymphatic channels. This however, does not explain the elevated levels of hydrogen found in the cysts [3]. The pulmonary theory suggests that alveolar rupture results in dissection of air along vascular channels in the mediastinum, tracking caudally to the retro peritoneum and then to the mesentery and bowel [16]. However, lack of interstitial emphysema within the lung or in the mesentery of many of these patients has led to scepticism amongst researchers [17]. The bacterial gas production theory is supported by reports of disappearance of gas with antimicrobial therapy. It is thought that bacteria reach the intramural compartment and produce gas or alternatively intraluminal bacterial production of gas promote gas diffusion across the bowel wall mucosa [3].

Patients with PI may present with chronic mild non-specific symptoms or may present acutely unwell with a surgical abdomen. A large study has shown symptoms in decreasing frequency of diarrhoea, bloody stools, abdominal pain, abdominal distension, constipation, weight loss, and tenesmus with rectal involvement [3]. PI secondary to other disorders is associated with signs and symptoms of that disorder. PI may be complicated by pneumoperitoneum, intestinal obstruction, volvulus and intestinal perforation.

The plain radiograph is the most common way to identify pneumatosis. In a review of 919 patients with PI proven by surgery or autopsy abdominal plain films were positive in two-thirds. [6] Circular collections of gas in the anatomical position of the bowel and its mesentery may be found [21]. Barium enema may also be helpful showing circumscribed attenuations in the contrast column or linear delineations along the margins [22]. Ultrasonography may also show bright echoes in the bowel wall and portal venous air [23]. Computed tomography is the best imaging modality for the diagnosis of PI, as denoted by the findings of intramural gas parallel to the bowel wall, having greater sensitivity than plain films or ultrsonograhphy [12,21]. More recently endoscopic ultrasound has also been suggested as a useful technique [26,27]

Treatment of PI is generally reserved for those with symptoms taking into account the whole clinical picture and any underlying conditions. Treatment with oxygen or hyperbaric oxygen has been shown to be effective. [28,29] This is based on the finding that the cysts of pneumatosis are filled predominantly with gasses other than oxygen, such as nitrogen. High oxygen tension in the inspired air leads to high partial pressure of oxygen in the inspired blood and concomitantly a low partial pressure of nitrogen which encourages resorption of nitrogen from the cysts [30,31] Other treatments including medical therapy with various antibiotics (metronidazole, tetracycline, ampicillin and vancomycin) and sclerotherapy have also been used with success. [5,35] a rare condition characterised by the presence of subserosal in resolution of cysts and symptoms [35].

In general, surgery is only indicated in symptomatic patients where medical therapy has failed and in patients with presenting acutely unwell with a surgical abdomen, especially those with signs of perforation, peritonitis or abdominal sepsis. Increased recognition of the benign form of PI has resulted in less unnecessary surgery, which in the past has occurred up to 27% of cases [5,6]. There have been numerous reports suggesting that a patient with a pneumoperitonuem with PI need not necessarily undergo a laparotomy unless clinically indicated as this is thought that the free air is due to rupture of an intramural bleb without true communication with the bowel lumen [[3], [4], [5], [6], [7], [8], [9], [10], [11], [12], [13], [14], [15], [16], [17], [18], [19], [20], [21], [22], [23], [24], [25], [26], [27], [28], [29], [30], [31], [32]].

## 4 Conclusion

Pneumatosis intestinalis is a rare condition that may manifest with a wide range of symptoms from mild abdominal pain to acute peritonitis. Its treatment is generally medical and even with radiological evidence of perforation laparotomy may not be indicated if the patient is clinically well.

## Conflictsof interest

There are no conflicts of interest including employment, consultancies, stock ownership, honoraria, paid expert testimony, patent applications/registrations, grants or other funding.

## Sourcesof funding

There are no sources of funding for this research.

## Ethical approval

This study is exempt from ethical approval in this institution.

## Consent

Written informed consent was obtained from the patients for publication of this case report and accompanying images. A copy of the written consent is available for review by the Editor-in-Chief of this journal on request.

## Author contribution

Dr Sunny Dhadlie.

- study concept

- data collection, analysis, interpretation

- writing the paper.

Contributers:

Dr Daniel Mehanna

- study concept

- Data collection, analysis, interpretation.

Mr James McCourtney

- data collection, analysis, interpretation.

## Registration of research studies

researchregistry4407.

## Guarantor

Dr Daniel Mehanna.

## Provenance and peer review

Not commissioned, externally peer reviewed.
